# Supplementary material for: Early Experience Analyzing Dietary Intake Data from the Canadian Community Health Survey—Nutrition Using the National Cancer Institute (NCI) Method
Source: Nutrients. 2019 Aug 15;11(8):1908. doi: 10.3390/nu11081908 (PMC6723340; doi:10.3390/nu11081908)
Supplement: Supplementary file 1 [file nutrients-11-01908-s001.pdf]

Flowchart for Estimation of Usual Intakes using the univariate NCI method

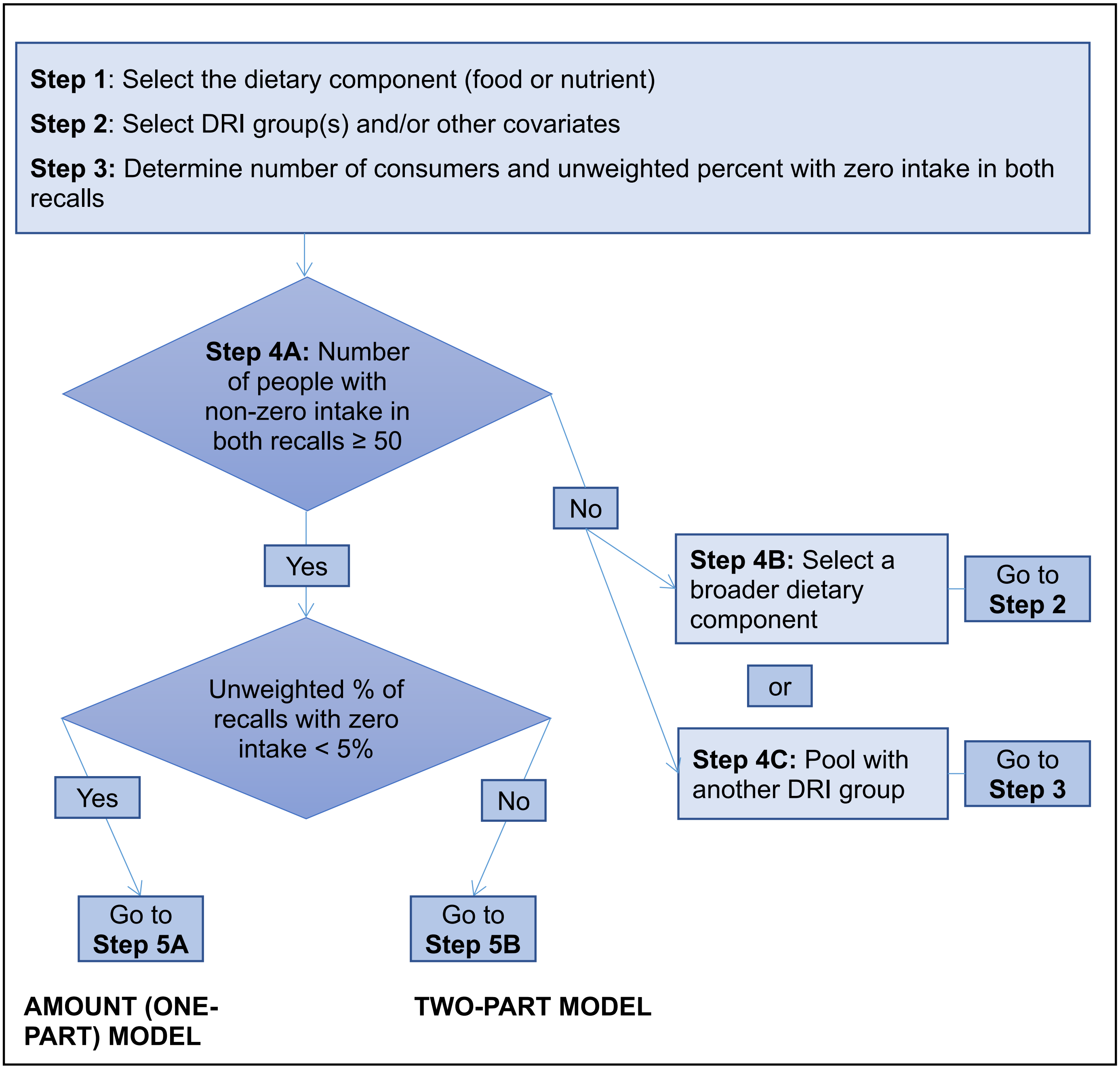

AMOUNT (ONE-PART) MODEL

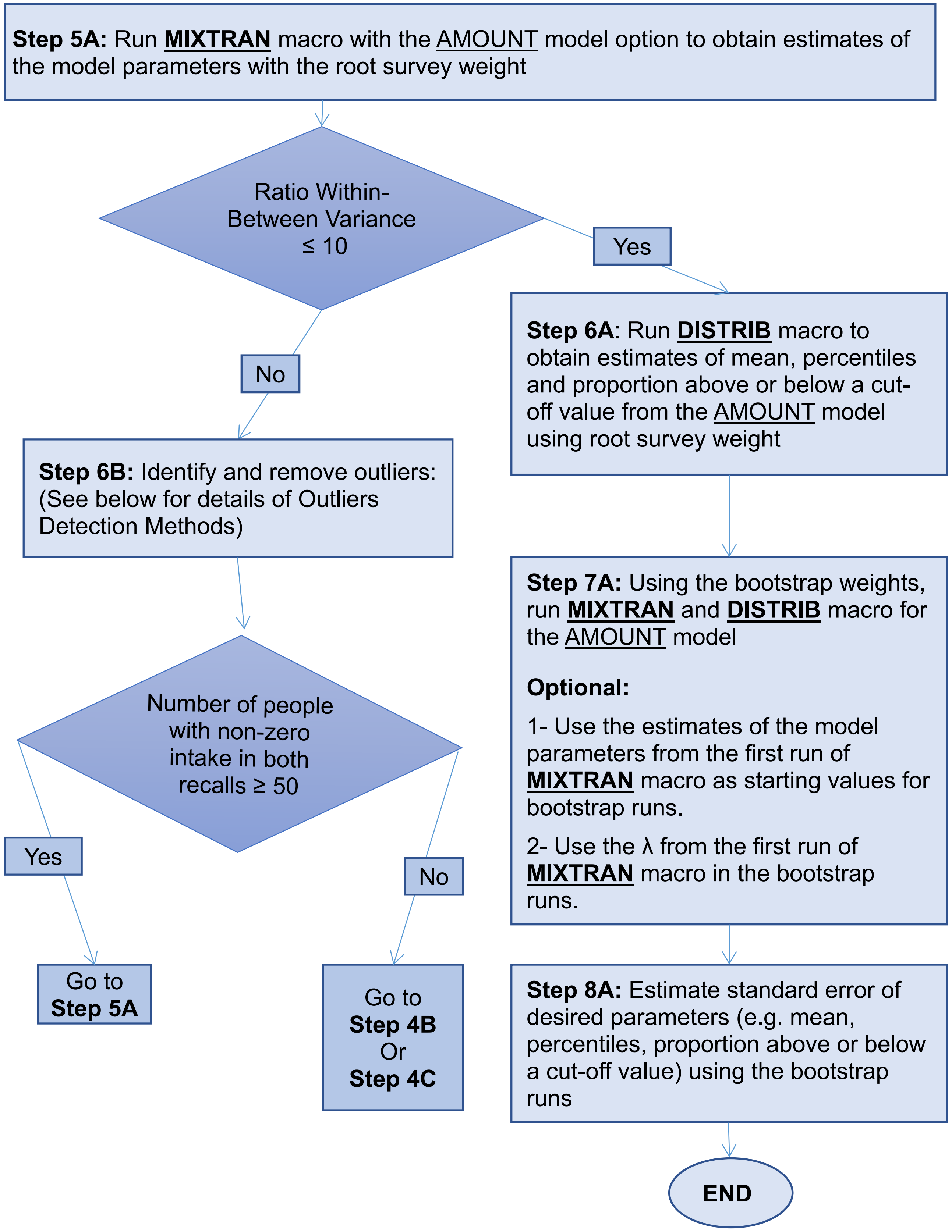

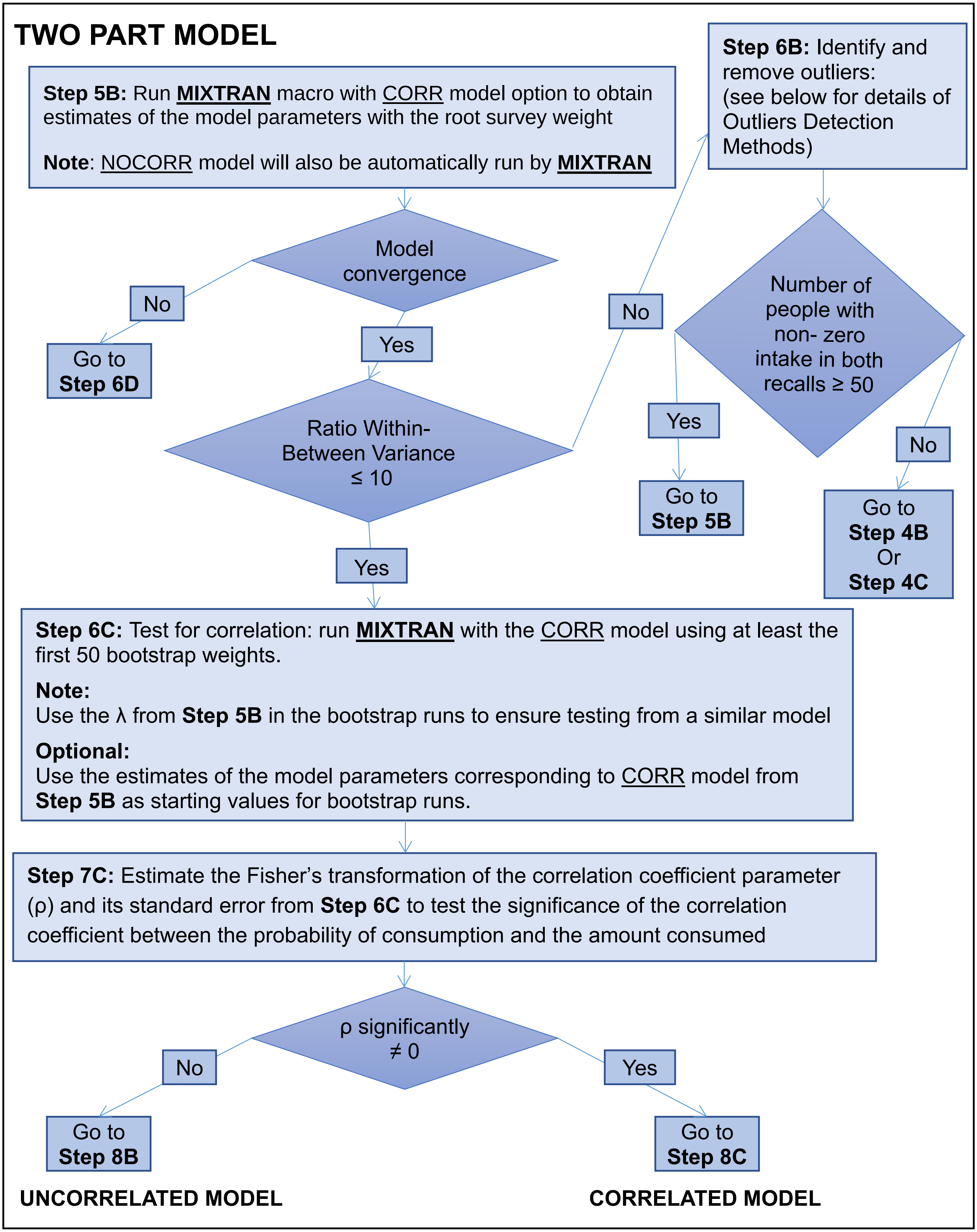
TWO PART MODEL
Step 5B: Run MIXTRAN macro with CORR model option to obtain estimates of the model parameters with the root survey weight
Note: NOCORR model will also be automatically run by MIXTRAN
Model convergence
No
Go to Step 6D
Yes
Ratio Within-Between Variance ≤ 10
No
Step 6B: Identify and remove outliers: (see below for details of Outliers Detection Methods)
Number of people with non- zero intake in both recalls ≥ 50
Yes
Go to Step 5B
No
Go to Step 4B Or Step 4C
Yes
Step 6C: Test for correlation: run MIXTRAN with the CORR model using at least the first 50 bootstrap weights.
Note:
Use the λ from Step 5B in the bootstrap runs to ensure testing from a similar model
Optional:
Use the estimates of the model parameters corresponding to CORR model from Step 5B as starting values for bootstrap runs.
Step 7C: Estimate the Fisher's transformation of the correlation coefficient parameter (ρ) and its standard error from Step 6C to test the significance of the correlation coefficient between the probability of consumption and the amount consumed
ρ significantly ≠ 0
No
Go to Step 8B
Yes
Go to Step 8C
UNCORRELATED MODEL
CORRELATED MODEL

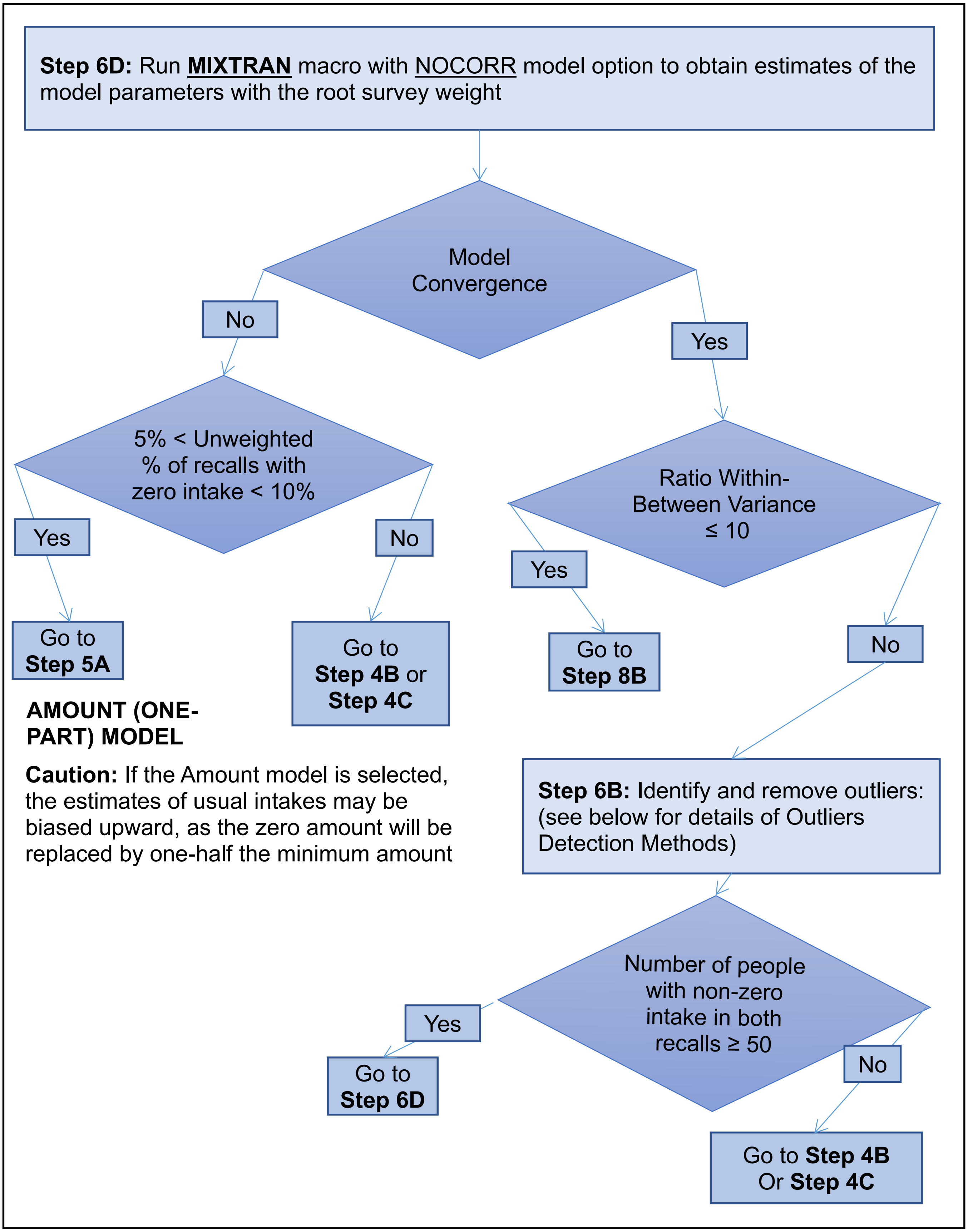

Step 6D: Run **MIXTRAN** macro with NOCORR model option to obtain estimates of the model parameters with the root survey weight
Model Convergence
No
Yes
5% < Unweighted % of recalls with zero intake < 10%
Yes
No
Go to **Step 5A**
Go to **Step 4B** or **Step 4C**
Ratio Within-Between Variance ≤ 10
Yes
No
Go to **Step 8B**
AMOUNT (ONE-PART) MODEL
Caution: If the Amount model is selected, the estimates of usual intakes may be biased upward, as the zero amount will be replaced by one-half the minimum amount
Step 6B: Identify and remove outliers: (see below for details of Outliers Detection Methods)
Number of people with non-zero intake in both recalls ≥ 50
Yes
No
Go to **Step 6D**
Go to **Step 4B** Or **Step 4C**

UNCORRELATED MODEL

Step 8B: Using parameter estimates from **MIXTRAN** corresponding to the NOCORR model option, obtained either as part of the **Step 5B** or from **Step 6D**, run **DISTRIB** macro to obtain estimates of mean and percentiles with the NOCORR option in the MODELTYPE parameter using the root survey weight.

↓

Step 9B: Run **MIXTRAN** and **DISTRIB** macros with the NOCORR model option using bootstrap weights (analysis of **Step 6C** may need to be re-run)

Optional:

1- Use the estimates of the model parameters from the **MIXTRAN** macro, obtained either as part of the **Step 5B or** from **Step 6D**, as starting values for bootstrap runs.

2- Use the λ from the first run of **MIXTRAN** macro in the bootstrap runs.

↓

Step 10B: Estimate standard error of desired parameters (e.g. mean, percentiles, proportion above or below a cut-off value) using the bootstrap runs

↓

END

CORRELATED MODEL

Step 8C: Using parameter estimates from **MIXTRAN** corresponding to the CORR model option, obtained from **Step 5B**, run **DISTRIB** macro to obtain estimates of mean and percentiles with the CORR model option using root survey weight

↓

Step 9C: Run **MIXTRAN** macro with the CORR model option with remaining bootstrap weights from **Step 6C.**

Note:

Use the λ from **Step 5B** in the bootstrap runs to be consistent with **Step 6C**

Optional:

Use the estimates of the model parameters corresponding to CORR model from **Step 5B** as starting values for bootstrap runs.

↓

Step 10C: Run **DISTRIB** macro with the CORR model option for all the bootstraps considered in **Step 6C** and **Step 9C**.

↓

Step 11: Estimate standard error of desired parameters (e.g. mean, percentiles, proportion above or below a cut-off value) using the bootstrap runs.

↓

END

For Step 6B: Methods for Outlier Detection used in Analysis of Nutrients and Episodically Consumed Foods

Method I: Large Within-Between Variance Components

- When the ratio of within/between variation is greater than 10, consider the mean distribution of the difference between Day 1 and Day 2 recalls.
- Values were identified as possible outliers if they fell ±3, ±2.5 or ±2 SD away from the mean distribution of difference between Day 1 and Day 2 values
- Day 2 recalls were removed as Day 1 recalls are considered to be less biased
- The scenario which first resulted in the within-between variance ratio less than 10 and excluded the fewest second 24hr recalls was retained

Method II: Normality Violation (Krebs-Smith [2])

- Perform a Box-Cox transformation of the raw non-zero values to approximate normality
- Extreme values identified as points either below the 25th percentile minus 2.5*IQR of the transformed distribution OR above the 75th percentile plus 2.5*IQR of the transformed distribution
- Analysis with and without extreme values conducted and compared
